# Obesity, Inflammation, and Lung Injury (OILI): The Good

**DOI:** 10.1155/2014/978463

**Published:** 2014-05-11

**Authors:** Cheryl Wang

**Affiliations:** ^1^Department of Anesthesiology, University at Buffalo, State University of New York, 3435 Main Street, Buffalo, NY 14214, USA; ^2^Veterans Affairs Western New York Healthcare System, Buffalo, NY 14215, USA

## Abstract

Obesity becomes pandemic, predisposing these individuals to great risk for lung injury. In this review, we focused on the anti-inflammatories and addressed the following aspects: adipocytokines and obesity, inflammation and other mechanisms, adipocytokines and lung injury in obesity bridged by inflammation, and potential therapeutic targets. To sum up, the majority of evidence supported that adiponectin, omentin, and secreted frizzled-related protein 5 (SFRP5) were reduced significantly in obesity, which is associated with increased inflammation, indicated by increase of TNF**α** and IL-6, through activation of toll-like receptor (TLR4) and nuclear factor light chain **κ**B (NF-**κ**B) signaling pathways. Administration of these adipocytokines promotes weight loss and reduces inflammation. Zinc-**α**2-glycoprotein (ZAG), vaspin, IL-10, interleukin-1 receptor antagonist (IL-1RA), transforming growth factor **β** (TGF-**β**1), and growth differentiation factor 15 (GDF15) are also regarded as anti-inflammatories. There were controversial reports. Furthermore, there is a huge lack of studies for obesity related lung injury. The effects of adiponectin on lung transplantation, asthma, chronic obstructive pulmonary diseases (COPD), and pneumonia were anti-inflammatory and protective in lung injury. Administration of IL-10 agonist reduces mortality of acute lung injury in rabbits with acute necrotizing pancreatitis, possibly through inhibiting proinflammation and strengthening host immunity. Very limited information is available for other adipocytokines.

## 1. Introduction

One-third of the adult Americans are obese and 2/3 are overweight [1, 2]. Obesity increases histrionically and is becoming pandemic worldwide in the past decades, predisposing these populations to great risk for gastric esophageal reflux disease (GERD) and subsequent aspiration pneumonia, asthma, obstructive sleep apnea syndrome (OSAS), and related comorbidities and mortality in lung injury, such as acute lung injury (ALI) and ARDS (acute/adult respiratory distress syndrome), even after being adjusted for other risk factors [3–6]. This remains true in a large array of studies in patients with ALI and critical conditions [7–9]. However, there were controversial reports as well.

Lung injury is a debilitating disease, with mortality close to that of breast cancer, costing our federal government at least 850 million dollars every year [10, 11]. This places a huge burden to our government as well as the suffering families. As the prevalence of obesity and its comorbidities increases skyrocketing, obesity related lung injury rises significantly in the past decades.

This may be mediated by depletion of the antioxidants, destroyed lung endothelium, reduced lung volume and chest wall compliance, and increased susceptibility of the lung to injury [12, 13]. Under obese state, there are changes with fat sites and sizes. Furthermore, obesity is a chronic systemic inflammatory process, with infiltration of macrophages and other cells. This inflammatory process is driven by the adipocytokines derived from adipocytes, macrophages, and other cells in adipose tissues, which cause an unbalance between the proinflammatory adipocytokines such as lepin, resistin, vasftin, and TNF*α* and the anti-inflammatory adipocytokines such as adiponectin, omentin, SFRP5, vaspin, ZAG, and interleukin-10 (IL-10) [14]. This process is accompanied by the polarization of macrophages, from “healthy” M2 to “unhealthy” M1 macrophages and the transformation of T helper (Th) cells from “beneficial” Treg and Th2 to “harmful” Th17 and Th1. These form an inflammatory soup, heavy with proinflammatory adipocytokines, which further activates Toll-like receptor 4 (TLR4), NF-*κ*B, and other signaling pathways, initiating a cascade of inflammatory process [15].

Furthermore, these changes modulate host defense responses, namely, the innate and adaptive immunity [16], regulating the susceptibility of the lung for injury. When a variety of insults occur, such as ozone (O_3_), gastric acid and bacterial and nonbacterial particles [6], the lung may become more susceptible for injury, depending on the overall balance between the offense and defense, the proinflammatory and anti-inflammatory adipocytokines.

Yet, limited articles have a comprehensive review of the overall balance of these adipocytokines and their relationship to the pathogenesis of lung injury. In our series of review articles, we will address these adipocytokines and their relationship with lung injury as the good, the bad, and the ugly: the anti-inflammatory (the good), the proinflammatory (the bad) and their impact on host defense response, and the immunity (the ugly). These contents will be included in three respective review articles, with the major objective to get a better view of the pathogenesis of lung injury in obesity, the molecular basis of other comorbidities in obesity, the research gaps in OILI, and the scientific and therapeutic targets in a more comprehensive and efficient fashion. And thus this important information will direct our research and scientific focus and further personalized medicine in this huge population in the near future.

**Figure 1:**
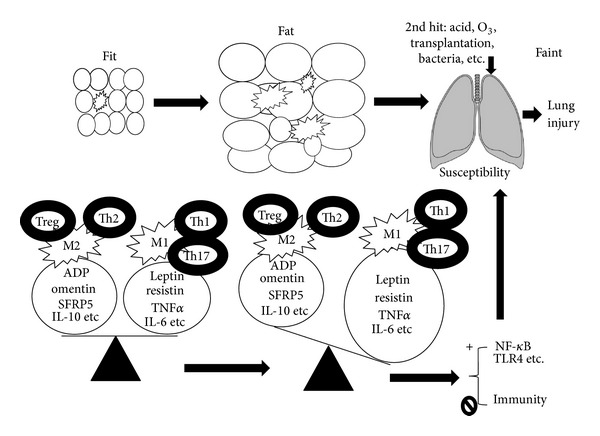
Fit-fat-faint: the overall mechanism of obesity, inflammation, and lung injury. In fit individuals, small fat cells secret proinflammatory and anti-inflammatory adipocytokines. There are balances between these adipocytokines, macrophages M1 and M2, T helper cells Th1 and Th2, and Th17 and Treg. Under fat state, fat cells got larger and infiltrated by more macrophages and other cells, secreting more proinflammatory adipocytokines and causing an unbalance between proinflammation and anti-inflammation. These activate NF-*κ*B and TLR4 signaling pathways and decrease host immunity, thus increasing susceptibility of the lung. When the 2nd hit occurs, such as aspirated acid under obesity or debilitated conditions, O_3_ in the air, bacteria, and surgeries, it is easier for the susceptible lung to get injured (faint). The final outcome depends on the overall balance. ADP: adiponectin.

In this review article, by reviewing the articles with animal models and preclinical trials as well as the clinical trials in human being related to OILI, we will focus on the anti-inflammatory adipocytokines (the good) and address from the following aspects: adipocytokines and obesity, inflammation and other mechanism involved, adipocytokines and lung injury in obesity bridged by inflammation, and some therapeutic potentials. The studies on obesity and inflammation will be addressed and summarized. Those related to lung injury will be discussed in detail. Some possible mechanisms involved are illustrated in Figure 1 and this review article will be summarized in Table 1.

## 2. Obesity, Inflammation, and Lung Injury: The Good

A large array of adipokines, cytokines, chemokines, and other factors were derived from adipose tissues [17]. In this review article, we refer to them as adipocytokines. Besides adipocytes, macrophage is believed to be a major contributor for these factors. The majority of the evidence supported that adiponectin, omentin, and SFRP5 are anti-inflammatory, the good, and are decreased in obesity, which is associated with increased systemic inflammation, indicated by increased circulating TNF*α*, C reactive protein (CRP), IL-6, and other proinflammatory cytokines/chemokines [17, 18]. Administrations of these adipocytokines promote weight loss and reduce inflammation [19]. Other anti-inflammatory adipocytokines beneficial for weight loss are ZAG, vaspin, IL-10, IL-1RA, TGF-*β*1, and GDF15 [20]. Yet, there were controversial reports.

Regretfully, very limited information is available for their roles in the pathogenesis of lung injury. We will do our best to get valuable information from these limited studies and discuss some possibilities.

### 2.1. Adiponectin

Adiponectin was first identified in adipocytes and highly conserved cross species [21–23]. It is also found in cardiomyocytes and skeletal muscle [24–27]. Adiponectin accounts for 0.01% of total protein in circulation, with a normal range of 2–20 *μ*g/mL, and is quickly cleared after secretion (half-life of 45 to 75 minutes) [28, 29]. Despite this fact, adiponectin concentration remains rather stable in plasma. A growing number of studies suggested that adiponectin is decreased in obesity and negatively correlated to visceral fat mass, inflammation, heart disease, injury, and many other diseases but positively to insulin sensitivity and promotes weight loss [30–33]. A positive correlation between adiponectin and fat mass at the lower extremities has been revealed but a negative one with that of the body trunk was typically seen in abdominal obesity. Furthermore, adiponectin drives fat deposit in small fat cells and subcutaneous adipose tissue but mobilizes visceral fat, supporting its beneficial effect in variety of organ injury, such as nonalcoholic fatty liver disease and fatty heart in obesity and T2DM. Administration of recombinant adiponectin or overexpression of adiponectin promotes weight loss increases insulin sensitivity and exerts anti-inflammatory effects [34]. There were controversial reports though [35–38].

**Figure 2:**
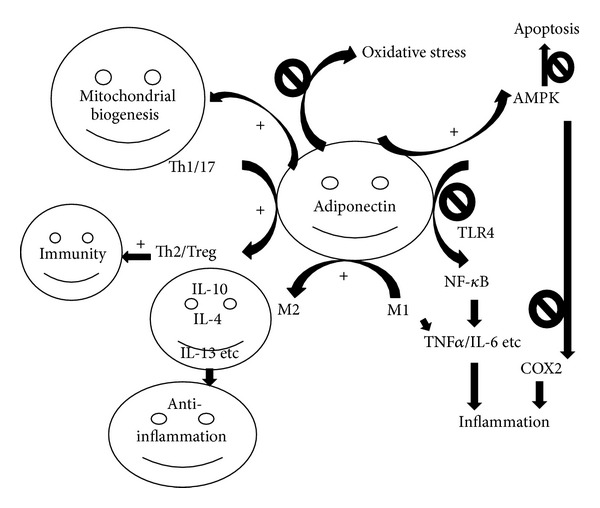
The major anti-inflammatory mechanism of adiponectin. Adiponectin polarizes macrophages from M1 to M2 and T helper cells from Th1 to Th2 and thus further increases immunity and has better anti-inflammatory effects. Furthermore, adiponectin activates AMPK and inhibits NF-*κ*B signaling pathways and thus inhibits inflammation. Additionally, adiponectin inhibits oxidative stress and stimulates mitochondrial biogenesis. Under obese state, the production of adiponectin is lower which is correlated with worse proinflammation and possible lung injury.

Figure 2 shows the major mechanism involved. Adiponectin decreases oxidative stress, inflammation, angiogenesis [39], apoptosis, and increases mitochondrial biogenesis [40], locally (paracrine/autocrine) and systemically (endocrine). In obesity, the unhealthy adipose tissues and infiltrated macrophages (more M1 than M2) [41] reduce the production of adiponectin and favorite proinflammatory process [42, 43]. It was suggested that adiponectin reduces inflammation and alleviates disease states, possibly through its suppression of TNF*α*, IL-6, and CRP and upregulation of IL-10 and IL-1RA [44–46]. Additionally, adiponectin increases mitochondrial density and biogenesis, adipocyte flexibility, and the host adaptation to stress [47]. The major signaling pathways involved are AMPK and PPAR*α*, PPAR*γ*, MEK-Erk, PI3K-Akt, APPL1, T-cadherin, Ca2+ and SIRT1, and so forth [40, 48–52], which promote fatty acid oxidation and glucose uptake into skeletal muscle and inhibit gluconeogenesis in liver.

Another important mechanism is the possible “polarizing effect” of adiponectin on macrophages and T helper cells. It was suggested that adiponectin may polarize macrophage from M1, proinflammatory state, to M2, anti-inflammatory state, as well as from “harmful” Th1/17 to “beneficial” Th2/Treg. This has been supported by both loss and gain of function studies [44, 53–58]. Moreover, adiponectin suppresses the proliferation of bone marrow-derived granulocyte and macrophage progenitors, inhibits phagocytic behavior of macrophages and proinflammatory cytokines secretion, and promotes anti-inflammatory cytokines of macrophages.

Adiponectin impacts host defense response and immunity, through inhibiting recruitment of leukocytes, increasing the remodeling of the lung, promoting phagocytosis of neutrophils and macrophages, modulating the productions of Th2 cytokines, and reducing/inhibiting B cell and natural killer (NK) cells in animal models [59]. Yet, little is known about the impact of adiponectin on host response in human beings, especially those related to lung injury. This is largely due to the difficulty in conducting large clinical and translational studies, as most of the patients are not in the conditions willing or able to be consented for these trials.

Adiponectin resembles the structures of complement factor C1q and surfactant proteins SpA and SpD of the lung, which function as pattern recognition molecules, and is possibly one major mechanism for adiponectin to limit the inflammation of the lung [60]. All three receptors of adiponectin, AdipoR1, AdipoR2, and T-cadherin, were detected in a variety of cells of the lung [61]. Furthermore, adiponectin can be transported from circulation to alveolar through T-cadherin on the endothelium. These support its potential roles in lung injury [62, 63]. Lung injury is a complicated pathogenesis process, including activation of immune system and inflammation, stimulation of endothelium, increased capillary permeability, neutrophil and macrophage infiltration, and leaking of albumin [64, 65]. The function of adiponectin in lung homeostasis is becoming a hot topic in the past few years, but it remains to be further determined and studied in more details. Recent data supported that obesity is a major risk factor for lung injury, and the adipose tissue derived adipokines and cytokines seem to play a very important role during this process [66–70]. This may be associated with activation and polarization of macrophages, stimulation of AMPK and COX2, and its effect on endothelium [71, 72]. Although there were controversial reports [73, 74], most of the evidence supported that reduced adiponectin level is associated with increased morbidity and mortality in critical care patients, lung transplantation, emphysema, asthma, chronic obstructive pulmonary disease (COPD), and acute lung injury of other causes [75–77], in animal models as well as in human beings. These were accompanied by macrophages activation, reduced clearance of apoptotic cells, and perivascular and lung inflammation [78, 79]. Moreover, administration of adiponectin improves outcome for asthma [80]. Additionally, in those contradicted reports mentioned above, adiponectin concentrations were tested during the critical illness, suggesting the possibility of the upregulation of adiponectin due to adaptation over time. This speculation was supported by the studies showing that increased adiponectin level and amelioration of the disease in mice with lupus when treated with PPAR*γ* agonist [81], regardless of the already elevated adiponectin level in these mice. From this aspect, we could hypothesize that the changes of adiponectin may be more important than its actual concentration during critical illness. In another word, administration of adiponectin may still benefit these patients regardless their elevated adiponectin level. If this is associated with upregulated receptor or other mechanism, it remains unclear. This being said, it is not difficult to understand the controversial results in patients with COPD. In patients with COPD, due to the long-term hypoxia and human body adaptation, adiponectin concentrations may be high or low, depending on how long and how badly the patients were sick and how the human body is adapting. With similar theory, those different results in patients with critical illness (e.g., those from APACHE II) or bacterial pneumonia seem reasonable as well. After all, the human body is an elegant system with delicate regulations. One cytokine/protein up or down simply cannot tell the whole story. The one-fit-all medicine is far from enough. Apparently, studies investigating the relationship of the changes of adiponectin and clinical outcomes, how the human body adapts, and what the host responses are would possibly provide more valuable information for clinical applications and further personalized medicine, both as a biomarker for a variety of diseases, severity, and prognosis and as a therapeutic potential.

Interestingly, it was found that total adiponectin levels and its active form, high molecular weight (HMW) isoform, are lower in men than their peer women at children-bearing age, which seems be associated with the high testosterone level in men [82].

Overall, adiponectin promotes anti-inflammation through inhibiting proinflammatory response, polarizing macrophages (from M1 to M2), and T helper cells (from Th1/17 to Th2/Treg), inhibiting TLR4-mediated NF-*κ*B activation, and protecting endothelium, suggesting that obesity may prime lung toward proinflammatory condition and more susceptible for injury due to hypoadiponectinemia, at least partially. Yet, the detailed mechanism remains to be further explored. Not much clinical data is available at this point.

Many drugs exert their effect via adiponectin and its receptors, decreased ceramide [46], and antiapoptotic sphingosine-1-phosphate (S1P), via its impact on insulin sensitivity and anti-inflammatory effects. This suggested that adiponectin could be a potential therapeutic target in obesity, metabolic syndrome, and its comorbidities, all of which are regarded as inflammatory processes. Yet, it remains unclear if adiponectin can be a potential therapeutic target for lung injury in human subjects. With the newly synthesized adiponectin receptor agonist, ADP355, and the defined adiponectin receptors in the lung, the role of adiponectin in the aforementioned inflammatory states, and its function as pattern recognition molecules, we expect that ADP355 would significantly benefit patients with obesity related lung injury. Apparently, more preclinical and clinical trials are warranted in the near future, for its function, mechanism, and potential therapeutic and preventive applications. Particularly, as adiponectin promotes weight loss and reduces inflammation and has receptors in the lung, studies targeting its role in OILI would be greatly beneficial for these populations. Both observational trials and therapeutic trials are largely needed.

### 2.2. Omentin

Omentin was initially found in intestinal cell (called intelectin) and then omental adipose tissue and human adipocytes (especially stromal vascular cells of visceral adipose tissue), but it is also expressed in lung, heart, placenta, and ovary [18, 83]. There are two forms, omentin 1 and omentin 2, which share 83% of amino acid sequences. Omentin 1 is rather more studied than omentin 2. In this article, we refer to omentin 1 as omentin. It was suggested that omentin level was lower in obese subjects, which is inversely associated with body mass index (BMI) and insulin resistance and positively with HDL and adiponectin [84]. Moreover, treatment for obesity with bariatric surgery or metformin increases serum level of omentin, which is associated with weight loss and improved insulin sensitivity, possibly through activating Akt signaling pathway. Studies showed the similarity of omentin and adiponectin [85–87], especially the effect on weight loss, insulin sensitivity, and type 2 diabetes (T2DM) [17, 88–92]. It was also reported that omentin level is low in Crohn's disease, synovial fluid of patients with rheumatoid arthritis, polycystic ovary syndrome (PCOS), and other inflammatory diseases [90, 93, 94]. Paradoxically, one recent study showed that increased omentin level was associated with nonalcoholic fatty liver disease (NAFLD), the very common comorbidity in obesity and T2DM [95]. As obesity, T2DM and NAFLD were all regarded as inflammatory process; these contradicted results may indicate an adaptation response. As shown in some studies with adiponectin, treating patients with NAFLD may still increase omentin level as well as reducing inflammation. Further studies are warranted to elucidate this phenomenon, the possible mechanism, and the changes with intervention.

**Figure 3:**
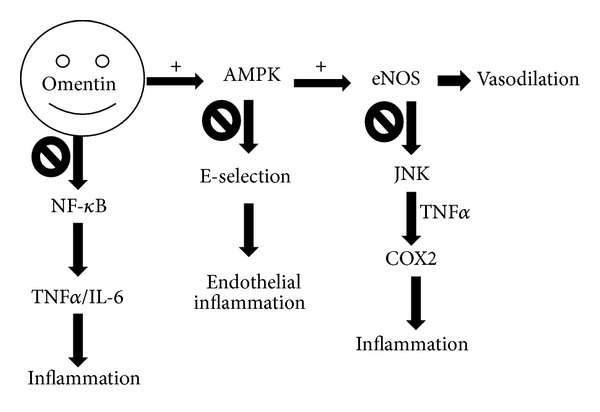
The anti-inflammatory mechanism of omentin. Omentin activates AMPK, which further blocks E-selection and reduces endothelial inflammation. AMPK also activates eNOS, which has vasodilation effect and blocks JNK signaling. JNK activates inflammation through TNF*α* mediated COX2 effect. Moreover, omentin inhibits NF-*κ*B signaling pathway and thus inhibits inflammation. Under obese state, the production of omentin is lower which is associated with worse proinflammation and possible lung injury.

As shown in Figure 3, omentin activates AMPK and eNOS, blocks Akt pathways, inhibits CRP, TNF*α*, and NF-*κ*B signaling pathways, reduces adhesion molecules, and thus has anti-inflammatory effect on smooth muscle cells and endothelium [96–99]. Administration with recombinant human omentin inhibits TNF*α*, decreases inflammation, and dilates vascular vessels, suggesting its potential therapeutic role in inflammation related conditions [100]. No study has assessed the possible impact of omentin on host defense response or immunity.

Three studies were conducted in patients with obstructive sleep apnea syndrome (OSAS) [101–103]. Two reported that omentin was elevated in patients with OSAS [103]. One was performed in Turkey and the other was in Germany. Both had rather small sample size. Another study was conducted in Chinese subjects and had a large sample size. It indicated that decreased serum omentin-1 levels could be regarded as an independent predictive marker for the presence and severity of OSAS. Omentin, the former called intelectin-1, is expressed in the lung. It was reported that intelectin-1 was secreted from malignant pleural mesothelioma and can be detected in pleural effusion, suggesting that it can be a biomarker for this malignancy. Furthermore, intelectin is needed for MCP-1 production in lung epithelium and causes airway inflammation in mice with asthma. If the receptor can be further determined, one may test if these effects are through paracrine/autocrine besides endocrine. As OSAS and asthma are highly associated with obesity, inflammation, and lung injury, this may suggest the association of omentin and lung injury. Additionally, given the fact that omentin blocks proinflammatory cytokines TNF*α*, and signaling pathway NF-*κ*B, it may be protective in lung injury. Furthermore, considering the similarity of omentin and adiponectin, we hypothesize that omentin exerts anti-inflammatory effect in lung injury. However, the possible proinflammatory effect of omentin may not be ignored as well. With the availability of recombinant human omentin, it would be greatly helpful to know if there are receptors for omentin in the lung, if omentin is anti-inflammatory in lung injury, and if omentin exerts its effect through adiponectin or independently, all of which may direct the therapeutic development in OILI and other related diseases.

### 2.3. SFRP5

SFRP5 was first discovered in adipocytes couple of years ago and the data was published in science [104]. In this study, it was shown that SFRP5-deficient mice fed on high-fat diet aggravated fat accumulation, inflammation, and systemic oxidative stress. Administration of SFRP5 reduced inflammation and attenuated insulin resistance, through decoying WNT mediated JNK activation in macrophages and adipocytes, and thus has systemic effects. Overexpression of SFRP5 promotes adiponectin and decreases TNF*α*, IL-6, and MCP-1, suggesting its anti-inflammatory effect. A recent study in Chinese subjects showed that SFRP5 is low in patients with T2DM. Furthermore, calorie restriction in obese subjects promoted weight loss and increased insulin sensitivity, which is correlated with improved SFRP5 level [105]. There were controversial reports. One recent study showed that SFRP1 but not SFRP 2–5 was found to be decreased in obesity and this is associated with insulin resistance [106]. However, in this study, it did show that SFRP1 increased adiponectin and reduced IL-6 and MCP-1 levels, which is consistent with the previous studies. Other isoforms should be further tested. Perhaps, it is the ratio of SFRP5 to other isoforms that matters. Another contradicted study also showed increased SFRP5 expression in diet-induced obesity [107]. In this study, the authors argued that this might be due to the fact that SFRP5 inhibits WNT signaling pathway and thus suppresses adipocytes mitochondrial metabolism and promotes oxidative stress. Combed with the previous data, it is confirmed that SFRP5 exerts its effect via inhibiting WNT signaling. This brought up the possibility that the isoforms of SFRP may vary cross species and ethics groups. Furthermore, the WNT at different compartments has different effects, which may partially explain these controversial results. Apparently, more studies are warranted.

**Figure 4:**
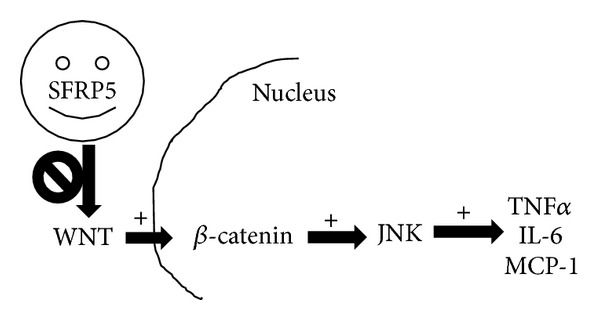
Signaling pathway of SFRP5, a decoy for WNT signaling pathway, which further activates *β*-catenin and then JNK. Activated JNK promotes proinflammatory cytokines TNF*α*, IL-6, and MCP-1. Under obese state, the production of SFRP5 was reduced and thus the decoying effect was weak, which is translated into the increased proinflammation and insulin resistance.

As shown in Figure 4, SFRP exerts its effects mainly through inhibiting WNT and JNK signaling pathways, which further inhibits the production of proinflammatory cytokines TNF*α*, IL-6, and MCP-1, and so forth. One recent study suggested that SFRPs might promote or suppress Wnt/*β*-catenin signaling, possibly depending on its receptors [108]. Additionally, SFRP5 regulates p53 and is a Hedgehog target to confine canonical WNT signaling. No information is available about its impact on host immunity and defense response.

Few studies were done in lung diseases. Limited information suggested that SFRP5 was low in pleura mesothelioma, and methylation of SFRP5 was associated with overall survival of lung cancer. Patients with unmethylated SFRP5 are more likely to benefit from EGFR-TKI therapy in non-small-cell lung cancer [109–111]. Based on its role in obesity and inflammation, we expect that SFRP5 exerts anti-inflammatory effect in obesity related lung injury. But it may depend on the compartments, the species, the ethnic groups, and other factors. With the availability of the recombinant SFRP5, more preclinical and clinical trials were needed to explore the effect of SFRP5 on OILI, as well as other comorbidities of obesity.

### 2.4. Vaspin

Vaspin is visceral adipose tissue-derived serpin (serpinA12) [112], and it is also rich in hypothalamus, skin, stomach, and subcutaneous adipose tissues [113]. Vaspin level is low in obesity, insulin resistance, and type 2 diabetes and increases with the attenuation of these conditions [114]. Furthermore, administration of vaspin suppresses leptin, TNF*α*, and resistin, reduces food intake, and improves glucose control and insulin sensitivity in obesity [115]. Yet, two recent studies with bariatric surgery in obese subjects revealed that vaspin decreased after surgery [116, 117], and the reduction was associated with leptin, HbA1c, and insulin sensitivity. These results were consistent with those treated with metformin [118]. This may suggest that there is a period of adaptation. Apparently, more detailed studies are needed to illustrate the time and impact of vaspin changes. Furthermore, vaspin was elevated in ulcerative colitis [119] and other inflammatory conditions, suggesting that it may exert proinflammatory effect as well. It was shown that vaspin is associated differently with metabolic syndrome in males and females, indicating its potential interaction or regulation by sex hormones [120]. This remains true in a variety of ethnic groups. Yet, these are observed phenomenon and the mechanism remains to be determined in detail.

Although the mechanism is largely unknown, it has been shown that vaspin inhibits vascular smooth muscle cells proliferation through inhibiting reactive oxidative species (ROS), MAPK, PI3K/Akt, and NF-*κ*B signaling pathways [121]. One recent study suggested that the inhibition of vaspin on ROS may be through NADPH oxidase [122], which is part of mechanism for cardiovascular disease (CVD). A cell membrane glucose-regulated protein (GRP78) was identified and regarded as a liver-specific receptor for vaspin, suggesting its potential role in liver diseases. No information is available about its impact on host immunity and defense response.

One study showed that high body fat mass with low cardiorespiratory fitness may be associated with increased vaspin in Korean population [123], suggesting its possible role in lung. No receptor for vaspin was defined in lung yet. As vaspin inhibits ROS and NF-*κ*B signaling pathways, activating AMPK and Akt pathways, along with its inverse relationship with respiratory fitness, we believe that vaspin may have a protective role in lung injury, through its anti-inflammatory effect. The important information would be to identify if there is a receptor for vaspin in the lung, if there is paracrine/autocrine effect of vaspin in lung, if the changes of vaspin is associated with less or worse lung injury in obesity, and if administration of vaspin attenuate lung injury. Additionally, it is worth the effort to determine if weight loss increases vaspin and if this is correlated with ameliorated lung injury.

### 2.5. Zinc-*α*2-glycoprotein (ZAG)

ZAG is expressed in adipose tissue, liver, breast, prostate, and so forth. It was identified as a lipid mobilizer in patients with cancer cachexia and obese mice, mediated by *β*3 adrenoreceptor through activating cyclic AMP (cAMP) pathway, increasing energy expenditure and lipolysis [124–127]. ZAG was expressed in visceral and subcutaneous adipose tissue and presented in stromal vascular cells and mature adipocytes [128]. So far, the majority of the evidence supported that ZAG level is lower in obesity and insulin resistance in mice with genetic defect or fed on high-fat diet as well as in human beings, and that there is an inverse relationship of ZAG with BMI and insulin resistance [129, 130]. Treatment for obesity and insulin resistance with liraglutide for 12 weeks increased ZAG level [131], indicating that ZAG may have a similar pattern as adiponectin. Additionally, overexpression of ZAG promoted weight loss and increased insulin sensitivity, through stimulating fatty acid oxidation. However, some studies [132, 133] revealed higher ZAG level in serum and white adipose tissue of obese/overweight individuals, as well as patients with chronic kidney disease, suggesting a possibility of “ZAG resistance,” like leptin resistance. Furthermore, it appeared that ZAG exerts its function as a lipid mobilizer in cancer cachexia more significantly.

ZAG was downregulated by TNF*α* and other proinflammatory cytokines in obesity, suggesting that its pattern is similar to that of adiponectin [128, 134]. Furthermore, studies in patients with CKD showed that ZAG is negatively correlated with TNF*α* and VCAM-1, suggesting its inverse relationship with systemic inflammation [135]. A negative association of reduced ZAG and increased CRP or MCP-1 was also reported in obesity, insulin resistance, and metabolic syndrome [136, 137]. Recent studies also demonstrated a positive correlation between ZAG and adiponectin and a negative one with leptin in human subjects [138]. It is possible that ZAG may act in paracrine/autocrine manner and facilitate adiponectin secretion from adipocytes.

Yet, very limited information is available for its relationship with lung injury. Based on the aforementioned, we think that ZAG may have anti-inflammatory effect on a variety of diseases, including lung injury. Considering its lipid mobilization in cancer, it may be valuable to find out what ZAG does in lung cancer, and if this is associated with the prognosis and clinical outcomes. But one may have to consider the possible “ZAG resistance.” Moreover, the fat mobilizing effect of ZAG was mediated by *β*3 adrenergic receptor, indicating its potential role in thermogenesis. Thus, it may be a therapeutic target in OILI. It would be greatly helpful if its receptor can be further identified. As the recombinant ZAG becomes available, both preclinical and clinical studies were needed to explore its function, mechanism, and potential therapeutic indications of ZAG.

### 2.6. IL-10

Interleukin-10 (IL-10) was initially identified as a product of Th2 cell and known as an anti-inflammatory cytokines inhibiting Th1 cell activity. It is derived from a variety of cells including monocytes, dendritic cells, lymphocytes, macrophages, and T cells. Although there were controversial reports, the majority of the evidence supported that IL-10 is negatively correlated to BMI, obesity, insulin resistance, and T2DM; furthermore, overexpression of IL-10 or administration of IL-10 reduces body weight, improves insulin sensitivity, and augments glucose control [139, 140].

**Figure 5:**
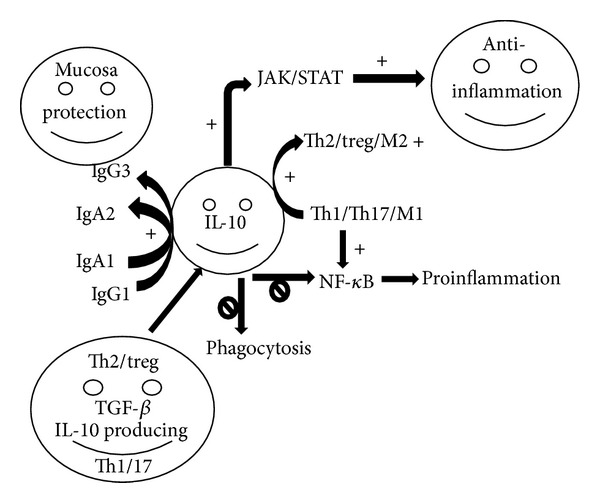
The anti-inflammatory mechanism of IL-10. IL-10 activates JAK/STAT signaling pathway, which further activates SCOS3 and anti-inflammatory process. It also polarizes Th1/Th17 to Th2/Treg and M1 to M2, which have anti-inflammatory effect. Moreover, it promotes the switches of IgG1 to IgG3 and IgA1 to IgA2, which have better mucosal protective effect. IL-10 also inhibits phagocytosis. IL-10 is reduced in obesity and this may contribute to the proinflammatory state and possible lung injury.

Figure 5 indicates the major mechanisms of IL-10. IL-10 polarizes macrophages from classically activated M1 to alternatively activated M2 phenotype and Th1/17 to Th2/Treg, upregulates IL-1 receptor and TGF-*β*, inhibits phagocytosis and proinflammatory cytokines and chemokines, which further blocks TLR4, NF-*κ*B, and other signaling pathways [15, 141, 142], and activates JAK/STAT signaling pathway. This results in decreased production of TNF*α*, IL-12, and other proinflammatory cytokines. Additionally, IL-10 is a switch factor for IgG1 and IgG3 and for IgA1 and IgA2, which has better protective effect for mucosa. Furthermore, therapy with mesenchymal stem cells (MSC) reprograms toward the polarization of macrophage M2 and increases IL-10 levels and thus has a protective role in sepsis, other infections, and acute lung injury [143].

Studies performed in lung transplantation showed that IL-10 decreases iNOS, IL-2, and TNF*α*, prevents ischemic-reperfusion injury, and inhibits acute rejection in animal models [144]. It was also proved that IL-10 protects lung from injury induced by LPS [145]. Early phase clinical trials suggested that IL-10 attenuates acute colitis [146], increases the tumor sensitivity of NK cells in rabbits with melanoma [147], promotes monocytes differentiating toward to tolerogenic DCs [148], and thus may have potential therapeutic value in autoimmune and transplantation related immune-compromised conditions. Interestingly, these studies suggested that only a small segment at C-terminal of IL-10 is responsible for its bioactivity. A synthetic IL-10 agonist, IT 9302, was administered to the rabbits with acute lung injury in acute necrotizing pancreatitis [149, 150]. It revealed that IT9302 reduced the mortality and the incidence of acute lung injury in rabbits with acute necrotizing pancreatitis, possibly by suppressing the productions of TNF*α*, IL-8, MCP-1, and adhesion molecule complex CD11b/CD18, as well as increasing serum IL-1*β* RA level. This is very encouraging, as most of the lung injury is related to inflammation and reduced immunity, such as OILI. In line with the aforementioned mechanism, along with the available agonists/analogues such as AM0010, SCH52000, RN1003, and IT9302, and its downstream signaling blockers such as CP-690 and CP-550, we hypothesized that IL-10 may have a protective role in lung injury, and more specifically, in acid aspiration induced lung injury in obesity. Related clinical trials are highly recommended to further define this, its bioactivity, safety, efficacy, and therapeutic indications.

### 2.7. Others: IL-1RA, TGF-*β*1, GDF-15, and So Forth

More adipocytokines showed anti-inflammatory effects on obesity and lung injury.

Interleukin-1 receptor antagonist (IL-1RA) was secreted naturally to encounter the effect of IL-1 and neutralize the proinflammatory effect of IL-1*β*, by competitively binding to IL-1 receptor I (IL-1RI). As it secrets at the time of IL-1 secretion, which is generally increased at the states of inflammation such as obesity, T2DM, and lung injury, it is understandable that IL-1RA is elevated in obese and diabetic subjects in Whitehall II cohorts [151] and a few other clinical trials. However, administration of recombinant IL-1RA (anakinra) lowers body weight and glucose level and decreases inflammation in patients with metabolic syndrome and T2DM [152, 153]. IL-1RA competitively binds to IL-1RI with IL-1 and thus decoys the inflammatory effects of IL-1. Deletion of IL-1RA leaves IL-1*β* unopposed and thus causes fetal inflammation systemically [154]. Under conditions with lung injury, IL-1 releases and triggers inflammation and IL-1RA releases to encounter this process. Administration of recombinant IL-1RA attenuates pulmonary fibrosis and pneumonia in animal models [155]. There are some ongoing/complete trials in subjects with rheumatoid arthritis, heart failure, pulmonary hypertension, diabetes, and other inflammatory conditions with recombinant IL-1RA anakinra. No ongoing/complete clinical trial in OILI was reported per the best of our knowledge.

TGF-*β* shows anti-inflammatory effect and has interaction with IL-10 [156, 157]. TGF-*β* is increased in obesity but overexpression of TGF-*β* inhibits adipogenesis [158]. Gene knockout of TGF-*β* confirmed its anti-inflammatory effect presented at the early stage and before the major attack of bacteria. Yet, these reports were controversial regarding its effect in obesity related lung injury. TGF-*β*1 has a very short half-life in circulation and this may contribute to these diverse results. TGF-*β*1 exerts its effect mainly through Smad signaling pathway. Some clinical trials with TGF-*β*1 antibodies such as GC1008, CAT-192, and LY2382770 are ongoing or complete in subjects with diabetes, diabetic kidney disease, and other inflammatory diseases. No ongoing/complete clinical trial in OILI was reported per the best of our knowledge.

GDF15, a member of TGF-*β* family, also known as macrophage inhibitory cytokine-1 (MIC-1), shares similarity with TGF-*β* [159, 160]. GDF15 increases in obesity but also suppresses food intake and reduces body weight in obese rodents [161]. GDF15 can be a biomarker for severity of lung diseases as well as inhibitor for cancer development [162]. No study was reported in OILI so far.

Although there are studies showing the anti-inflammatory effect of leptin, there are leptin receptors in lung, alveolar epithelium, and macrophages, and leptin plays very important roles in immunity and host defense response, especially for activation of cell mediated immunity, as leptin is regarded as a proinflammatory adipokine in obesity and lung injury, supported by the majority of the clinical trials and animal studies [59]. Thus, we include leptin in other papers and will not discuss much here.

## 3. Potential Therapeutic Targets

### 3.1. Adiponectin and Its Related Receptors

As addressed previously [19], due to the delayed discovery of the receptor for adiponectin, there is no clinical utilization of adiponectin. Yet, based on what we reviewed here, adiponectin showed a strong anti-inflammatory effect in obesity, through its activation of AMPK and stimulation of mitochondrial biogenesis, as well as its inhibition of NF-*κ*B signaling pathways and oxidative stress; we believe that adiponectin and adiponectin receptor agonist as well as AMPK activator would greatly benefit patients from a variety of aspects, including lung injury in obesity. With the availability of adiponectin receptor agonist, ADP355 [163], we expect that more preclinical and clinical interventional trials in OILI will be conducted. Someday, patients with OILI and other inflammatory diseases will be greatly benefited, especially those with obesity.

One major obstacle is the route and form of the agents. For lung injury, inhalation and intravenous injection or infusion would be appropriate. Details for getting the active molecule into the system and the modification after administration need to work out. Alternates would be other agents promoting adiponectin production, such as PPAR*γ* agonist, the market-available thiazolidinediones (TZDs), omega-3, and dietary modifications.

### 3.2. Omentin and Its Related Receptors

As the definitive receptor of omentin has not yet been identified in the lung, it is difficult to define the exact role of omentin in obesity related lung injury. More studies about its molecular and cellular mechanism are warranted for further advance. However, based on its inhibition to TNF*α*, IL-6, and other proinflammatory cytokines, its blocking on NF-*κ*B and TLR4 signaling pathways, its potential role in OSAS, as well as its association with inflammatory states such as Crohn's disease, rheumatoid arthritis, and PCOS, we believe that it favors anti-inflammation and may have therapeutic potential in obesity and its comorbidities including lung injury. Yet, most exploration of its therapeutic role is still in the preclinical stage, and there is no complete or ongoing clinical trial. With the availability of recombinant omentin, we believe that further studies from these aspects would provide valuable information in the near future.

### 3.3. SFRP5 and Its Related Receptor

Based on the effect of SFRP5 on weight loss, its signaling pathway, and the availability of the recombinant SFRP5, we expect more preclinical study and clinical trials in related area. As SFRP5 does reduce production of proinflammatory TNF*α*, IL-6, and MCP-1, we expect it to exert anti-inflammatory effect in obesity related lung injury.

### 3.4. IL-10 and Its Related Receptor

Based on the switch effects of IL-10 on macrophages, Th cells, IgG, IgA, and inhibition on proinflammation and Th1, IL-10 may have a great therapeutic potential in treating infections, inflammation, and related diseases including lung injury in obesity. As mentioned, synthetic interleukin-10 agonist such as IT9302 varnishes acute lung injury in rabbits with acute necrotizing pancreatitis [164] and promotes monocytes differentiation to tolerogenic DCs [165]. This suggested its therapeutic potential for autoimmune and transplantation-related disease, as well as its potential therapeutic benefit in OILI and other inflammatory diseases. Clinical trials with human synthetic interleukin-10 are still in the early phase, such as phase 1 trial with SCH 52000 in patients with Wegener's granulomatosis, phase 2 trial with RN1003 for scar reduction, phase 2 trial with recombinant human interleukin-10 for psoriasis, and phase 2 trial with Tenovil TM in prevention of post-ERCP acute pancreatitis. No ongoing or complete clinical trial for this agonist in OILI was reported. More trials in wider area with larger population are encouraged.

## 4. Summary and Research Gaps

As shown in Table 1, we sum up this review article as follows.

1. The majority of evidence supported that adiponectin, omentin, and SFRP5 were reduced significantly in obesity, which is associated with increased inflammation and possible lung injury, indicated by increase of TNF*α* and IL-6, through activation of TLR4 and NF-*κ*B signaling pathways.
2. Administration of these adipocytokines promotes weight loss and reduces inflammation.
3. IL-10, ZAG, vaspin, IL-1RA, TGF-*β*1, and GDF15 seem to be anti-inflammatory.
4. There were controversial reports, though.
5. Yet, there is a huge lack of studies for obesity related lung injury. Some groups investigated the effect of adiponectin on lung transplantation and subsequent changes for graft function, asthma, COPD, and pneumonia, supporting its anti-inflammatory effects and protective role. Synthetic IL-10 agonist reduces mortality of acute lung injury in rabbits with acute necrotizing pancreatitis, possibly through its inhibition of proinflammatory and promotion of anti-inflammatory adipocytokines, as well as its augmentation of host immunity. No study was performed in acid aspiration induced lung injury in obesity. More preclinical and clinical trials in wider area with larger population are warranted.
6. For other adipocytokines, there are very limited studies in obesity related lung injury.
7. In OILI, there is not much information available for clinical trials and translational research because most of the agonists were recently synthesized. Translational studies focusing on the mechanism should reveal valuable information for further investigation and therapeutic potentials. The early phase trials would need to focus on safety, efficacy, and bioavailability at this time point. In the near future, all kinds of related indications should be explored and determined.

## Figures and Tables

**Table 1:** Adipocytokines in obesity, inflammation, and lung injury: the good (trend).

Adipocytokine	Main functions*	Obesity	Inflammation	Lung injury	Agents available
Adipo-nectin	(1) Promotes weigh loss (2) Increases IS (3) Anti-inflammatory (4) Protects lung from injury	↓*	↓*	↓*	ADP355

Omentin	(1) Promotes weigh loss (2) Increases IS (3) Anti-inflammatory (4) Protects lung from injury	↓*	↓*	↓*	Recombinant

SFRP5	(1) Promotes weight loss (2) Increases IS (3) Anti-inflammatory (4) Protects lung from injury	↓*	↓*	↓*	Recombinant

Vaspin	(1) Decreases in obesity, T2DM, metabolic syndrome (2) Increases IS (3) Anti-inflammatory (4) No information in lung injury	↓*	↓*	?	Recombinant (OPPA00718)

ZAG	(1) Lipid mobilizer (2) Increases IS (3) Anti-inflammatory (4) No info in lung injury	↓*	↓*	?	Recombinant

IL-10	(1) Promotes weigh loss (2) Increases IS (3) Anti-inflammatory (4) Protects lung from injury	↓*	↓*	↓*	SCH52000 RN1003 IT9302 AM0010

IL-1RA	(1) Increases in obesity T2DM, metabolic syndrome, and lung injury (2) Encounters IL-1 and is anti-inflammatory	↑*	↑*	↑*	Recombinant (Anakinra)

TGF-*β*1	(1) Increases in obesity T2DM, metabolic syndrome, and lung injury (2) Anti-inflammatory	↑*	↑*	↑*	GC 1008 CAT-192 AP12009 LY2382770

GDF-15	(1) Increases in obesity, T2DM, metabolic syndrome, and lung injury; (2) Anti-inflammatory	↑*	↑*	↑*	Recombinant

*The majority of the evidence is supportive for this trend, but there were controversial reports. IS: insulin sensitivity. SFRP5: secreted frizzled-related proteins. IL: interleukin. ZAG: zinc-alpha2-glycoprotein. IL-1RA: interleukin 1 receptor antagonist. TGF: tumor growth factor. GDF: growth differentiation factor.
